# Firm foundation or neglected cornerstone? The paradox of Article 5.3 implementation and the challenge of strengthening tobacco control governance

**DOI:** 10.1136/tobaccocontrol-2022-057344

**Published:** 2022-03-08

**Authors:** Rob Ralston, Stella Bialous, Jeff Collin

**Affiliations:** 1 Global Health Policy Unit, The University of Edinburgh School of Social and Political Science, Edinburgh, UK; 2 Center for Tobacco Control, UCSF, San Francisco, California, USA

**Keywords:** tobacco industry, public policy, low/middle income country, global health

As well as being the first international public health treaty initiated and negotiated by the WHO, the WHO Framework Convention on Tobacco Control (FCTC) is the only such convention to explicitly seek to manage risks posed by a specified industry with opposing interests.^1 2^ The general obligation under Article 5.3 -requires states to protect the ‘setting and implementing [of] their public health policies with respect to tobacco control… from commercial and other vested interests of the tobacco industry’.^1^ This obligation is elaborated via implementation guidelines in eight recommendations and 34 subrecommendations,^3^ providing the basis for the FCTC’s distinctive model of health governance.^4^ Yet, recognition of the significance of Article 5.3 is accompanied by questions and concerns about its progress and status within the FCTC.^5–7^

Such questioning raises what might be termed the paradox of Article 5.3, which can be considered as having three parts. The first concerns the centrality of efforts to minimise industry interference to FCTC implementation. Civil society voices have emphasised Article 5.3 as ‘the backbone of the Convention’,^8^ while the FCTC’s official impact assessment saw strengthening adherence as ‘the single highest priority’ for advancing international tobacco control.^9^ Yet, Article 5.3 has also often seemed somewhat peripheral to core tobacco control interventions, exemplified in 2008 in its absence from the MPOWER package of six demand-side measures identified as the most important and effective.^10^

The second aspect considers the extent of its success as an innovation in health governance. Article 5.3 is cited as a model for managing conflict of interest with other unhealthy commodity industries, being presented as critical to tackling non-communicable diseases,^11^ key to developing effective governance for alcohol^12^ and as a precedent for excluding corporate interests in fossil fuels and agribusiness from environmental policymaking.^13^ This status seems hard to reconcile with the limited evidence base regarding the effectiveness of Article 5.3 measures at national level to date. While there are examples of good practice regarding specific measures,^14^ an extensive analysis of Party implementation reports, tobacco control laws and broader guidance for government officials found that only 16% of guideline recommendations have been implemented.^7^ The 2021 global progress report based on self-assessed country reports indicates that a majority of Parties (73%) adopted or implemented ‘some measures’ over the preceding 3 years,^15^ largely unchanged from the previous reporting period (72%).^5^ Civil society monitoring suggests that, in the context of the COVID-19 pandemic, management of industry interference has been deteriorating in more countries than have seen improvements.^16 17^

The third, and most challenging, dimension of this paradox lies in the combination of apparent simplicity from a tobacco control perspective with high levels of complexity when viewed in a broader governance context. The norms around excluding the tobacco industry from policymaking are perhaps so well established within the field that there can be frustration with implementation challenges, particularly with limited engagement from officials in non-health sectors. This is reflected in the sense that, given implementation guidelines, toolkits and resources and the consistency of industry tactics, then ‘addressing tobacco industry interference should be simple’.^18^

The intuitive appeal of Article 5.3 as embodying what seems obvious and necessary to many within tobacco control should not detract from how challenging it can be for many policymakers and government officials. The commitment to minimise policy interactions with the tobacco industry can run counter to models of private sector partnership and collaboration dominant in other policy areas^4^; require adaptation of standard daily operating procedures^19^; imply tensions with understandings of ‘good governance’ and expectations of stakeholder engagement^20^; and conflict with the mandates of government officials in other policy spheres.^21^

## Researching governance challenges

Across six case studies and two comparative analyses, this supplement is devoted to unpacking the challenges experienced by policymakers, officials and advocates in efforts to minimise industry interference. It aims to address the limited attention to date within public health research to institutional and organisational processes. Earlier articles have assessed Article 5.3 implementation using Party reports^7^ and explored how guideline recommendations have been subverted or ignored by governments working with the tobacco industry.^22 23^ These have been complemented by studies of intersectoral coordination^24–26^ and tensions across health and economic policy norms.^4 21 27^ There remains a significant need for qualitative research that explores policymakers’ perceptions and experiences of implementation in specific institutional contexts. Empirical and conceptual gaps persist in understanding Article 5.3 as a model and instrument of tobacco control governance, while the dearth of studies in low and middle-income country (LMIC) contexts is particularly striking.

The Tobacco Control Capacity Programme created an opportunity for a coordinated approach to examining such issues across diverse contexts.^28^ Article 5.3 was identified as a research priority by LMIC partner institutions and via stakeholder engagement, with research led by five project teams in Addis Ababa, Dhaka, Kampala, Delhi and Manipal, Karnataka. This was based on semistructured interviews with government officials, NGO officials and health advocates, employing a thematic interview guide that allowed adaptation to specific contexts.

The papers in this supplement begin to address empirical and conceptual gaps within public health research, highlighting persistent challenges in implementing Article 5.3 across disparate social, political and economic contexts. In their analysis of Article 5.3 and whole-of-government accountability in Uganda, Male *et al* 29 highlight substantial variations in awareness and engagement across government sectors. While Uganda’s 2015 Tobacco Control Act is regarded as a success story in advancing key Article 5.3 recommendations, this paper demonstrates how responsibility for minimising industry interference was widely seen as restricted to the Ministry of Health, with difficulties exacerbated by competing mandates across government agencies and perceived tensions with economic growth. In the context of Ethiopia, Hirpa *et al* 30 explore related issues around institutionalised practices of stakeholder consultation in policymaking. This contrasts Article 5.3 measures requiring the government to minimise interactions with the tobacco industry with persisting collaboration and engagement, reflecting both wider practices of consultation and the institutional legacy of Ethiopia’s previously state-owned tobacco monopoly.

Implementation within India’s federal political system is explored via two papers. Bassi *et al* 31 develop a comparative analysis of Article 5.3 initiatives across state and district governments, emphasising the importance of lesson drawing across jurisdictions and of technical support from civil society. This demonstrates the potential for subnational policy innovations in tobacco control governance, while acknowledging significant variations and gaps in coverage of Article 5.3 guideline recommendations. One such gap is the limited attention to denormalisation of tobacco industry corporate social responsibility (CSR), reflecting tensions with national legislation that requires large companies to undertake CSR initiatives. In the state of Karnataka, Kumar *et al* 32 explore tensions between tobacco industry CSR and district-level approaches to Article 5.3 implementation, arguing that scope to reconcile health, agriculture and commercial agendas has been constrained by promotion of tobacco producer interests at national level.

While those studies explore contexts that have seen substantive policy innovations, in Bangladesh Abdullah *et al* focus on the stalled development of proposed Article 5.3 measures.^33^ They demonstrate how institutional and individual conflicts of interest between government officials and tobacco companies have obstructed progress, with the National Tobacco Control Cell lacking the resources and political authority to break this impasse. The final case study is drawn from a linked research project on tobacco control in the UK Overseas Territories, exploring the complexities of managing conflict of interest in small island contexts.^34^ Given that such states constitute one-fifth of FCTC Parties, understanding the implications of their distinctive social, political and economic contexts assumes global significance.

Two comparative papers draw on interview data from multiple contexts to identify shared challenges and develop conceptual frameworks for understanding Article 5.3 implementation. In the first, Barry *et al* 35 critically assess shared coordination challenges across Uganda, Ethiopia, India and Bangladesh. This analysis points to barriers to horizontal coordination; ambiguity and uncertainty about responsibility for tobacco control and FCTC implementation; competing mandates; the limited capacity of coordination mechanisms; and explores vertical dimensions of coordination across local, state and national governments. The second comparative paper by Ralston *et al* 36 conceptualises Article 5.3 and its implementation guidelines as a policy instrument, drawing on data from Ethiopia, Uganda and India to examine Article 5.3 as comprising norms, rules and policy tools. This analysis differentiates between a core norm of a fundamental conflict with the tobacco industry and a governance norm that public health policies should be protected from industry interests, and explores their interaction with specific rules (such as restricting interactions with the tobacco industry) and policy tools (eg, guidance for public officials on managing such interactions).

## Implications and opportunities

There are limits to the generalisability of findings from a small number of case studies, and there remains a need for further qualitative research, including to explore opportunities to strengthen implementation via deliberative approaches with policymakers and by drawing on related initiatives in other fields.^37 38^ Yet, the research presented here does suggest ways in which the three aspects of the Article 5.3 paradox might be better understood.

Examining Article 5.3 as a policy instrument, for example, highlights broad success in establishing awareness and support for the norm of a fundamental conflict of interest with the tobacco industry, a norm that helps advance tobacco control even in jurisdictions not covered by the FCTC.^34^ This achievement explains the esteem in which Article 5.3 is held by those seeking to demonstrate a need to minimise policy interference by other unhealthy commodity industries, but presenting it as a model can convey an impression within commercial determinants of health debates that this problem has been solved in tobacco control.^11 13^ The scope of the remaining task is demonstrated by the limited development of rules and tools to define and manage interactions^36^; by mapping consistent gaps in coverage of guideline recommendations^31^; by highlighting the ‘lacuna’ under which engagement with industry CSR activities may not be seen as violating Article 5.3 guidelines^32^; and by demonstrating the challenge of managing close relationships arising from state interests in the tobacco industry.^30 33^

Questions concerning centrality of Article 5.3 to tobacco control and FCTC implementation efforts are also illuminated by recognising both the essential status of the principle of a conflict of interest and the limited development of policy mechanisms to actively manage interference. The significance of Article 5.3 has been articulated as that of ‘a cross-cutting issue’^39^ underpinning all tobacco control policies. But, as with other cross-cutting issues in health policy (notably health inequalities),^40^ progress is likely to be limited if this broad framing does not generate sufficient focused resources or active promotion. Similarly, Article 5.3 measures may sit in splendid isolation, with inadequate attention to ensuring that this underpinning function is best performed. Some important measures viewed as constituting best practice can have a narrow scope, such as applying only to health officials.^14 31^ This can risk perpetuating marginalisation and limited engagement by other government departments, and highlights the value of ensuring that measures are fully integrated with broader governance obligations across Article 5. This requires recognising that tobacco control objectives can be advanced via wider approaches to strengthening public sector governance across freedom of information, codes for government officials, tackling corruption and promoting transparency and accountability.^41^

Any sense that Article 5.3 implementation ought to be straightforward becomes hard to sustain when situated within the broader challenges of developing cohesive policy approaches across multiple government departments.^35^ The difficulties of promoting ‘whole-of-government’ or ‘joined up’ government responses to any issue with implications across diverse policy areas constitute a defining challenge for policymaking.^42 43^ Efforts to implement Article 5.3 further require that governments do so in highly polarised policy contexts, entailing adaptations to usual operating practices, amid seemingly conflicting mandates, confronted by powerful opposing interests and typically with limited resources and political commitment.

Tobacco control therefore needs to recognise that Article 5.3 can be perceived as challenging to officials working in other policy spheres, but it is also clear that its effective implementation is by no means an impossible task. This research demonstrates that measures adopted to implement Article 5.3 recommendations are capable of changing government practices; provide scope for bottom-up innovation at subnational levels that drive important changes; are being supported and shaped by active engagement with civil society; and crucially can attract support from diverse ministries and departments (though this requires more sustained efforts to actively reach out and engage).^35^

Importantly, the international context for supporting such efforts is in many respects increasingly favourable. Member states’ priorities for FCTC implementation now centre firmly on strengthening governance,^44^ the FCTC 2030 project has provided financial support to advance implementation in selected LMIC contexts,^45^ the Pan American Health Organization is developing an instrument to support states in monitoring progress on implementing mechanisms^46^ and collaboration with the United Nations (UN) Development Programme has increased understanding of how to promote multisectoral engagement.^41^ While centred primarily on industry monitoring, Bloomberg Philanthropies’ major investment in the Stopping Tobacco Organisations and Products (STOP) partnership^47^ generates unprecedented opportunities to raise awareness of and minimise industry interference, and innovative approaches to managing conflict of interest in other policy spheres create important opportunities for lesson drawing.^37 38^ Renewed efforts to implement Article 5.3 guideline recommendations hold the prospect of advancing FCTC implementation, enabling broader progress towards tackling commercial determinants of health^48^ and accelerating progress across UN Sustainable Development Goals.^49^ Realising such gains requires tobacco control to advance implementation in concert with such broader governance agendas; unpacking the paradox of Article 5.3 and developing a detailed understanding of the diverse governance challenges entailed is key to advancing such objectives.
